# Suggestive evidence of associations between liver X receptor β polymorphisms with type 2 diabetes mellitus and obesity in three cohort studies: HUNT2 (Norway), MONICA (France) and HELENA (Europe)

**DOI:** 10.1186/1471-2350-11-144

**Published:** 2010-10-12

**Authors:** Karianne Solaas, Vanessa Legry, Kjetil Retterstol, Paul R Berg, Kirsten B Holven, Jean Ferrières, Philippe Amouyel, Sigbjorn Lien, Javier Romeo, Jara Valtueña, Kurt Widhalm, Jonatan R Ruiz, Jean Dallongeville, Serena Tonstad, Helge Rootwelt, Bente Halvorsen, Marit S Nenseter, Kare I Birkeland, Per M Thorsby, Aline Meirhaeghe, Hilde I Nebb

**Affiliations:** 1 Department of Endocrinology, Oslo University Hospital, Aker, Oslo, Norway; 2 Department of Nutrition, Faculty of Medicine, University of Oslo, Oslo, Norway; 3 INSERM, U744; Institut Pasteur de Lille; Univ. Lille Nord de France; UDSL; Lille, France; 4 The Lipid Clinic, Oslo University Hospital, Rikshospitalet, Oslo, Norway; 5 Norwegian Medical Agency, Oslo, Norway; 6 Centre for Integrative Genetics, The Norwegian University of Life Sciences, Aas, Norway; 7 Department of Cardiology, Toulouse University School of Medicine, Rangueil Hospital, Toulouse, France; 8 Immunonutrition group, Department of Metabolism and Nutrition, Institute Frio-ICTAN, Spanish Scientific Research Council, Madrid, Spain; 9 Facultad de Ciencias de la Actividad Fisica y del Deporte- INEF, Universidad Politécnica de Madrid, Madrid, Spain; 10 Department of Paediatrics, Division of Clinical Nutrition and Prevention, Medical University of Vienna, Vienna, Austria; 11 Unit for Preventive Nutrition, Department of Biosciences and Nutrition at NOVUM, Karolinska Institutet, Stockholm, Sweden; 12 Department of Preventive Medicine, Oslo University Hospital, Ulleval, Oslo, Norway; 13 Department of Medical Biochemistry, Oslo University Hospital, Rikshospitalet, Oslo, Norway; 14 Research Institute for Internal Medicine, Oslo University Hospital, Rikshospitalet, Oslo, Norway; 15 Faculty of Medicine, University of Oslo, Norway; 16 The Hormone Laboratory, Oslo University Hospital, Aker, Oslo, Norway

## Abstract

**Background:**

The liver X receptors (LXR) α and β regulate lipid and carbohydrate homeostasis and inflammation. *Lxrβ*^*-/- *^mice are glucose intolerant and at the same time lean. We aimed to assess the associations between single nucleotide polymorphisms (SNPs) in *LXRβ *and risk of type 2 diabetes mellitus (T2DM), obesity and related traits in 3 separate cohort studies.

**Methods:**

Twenty *LXRβ *SNPs were identified by sequencing and genotyped in the HUNT2 adult nested case-control study for T2DM (*n *= 835 cases/1986 controls). Five tag-SNPs (rs17373080, rs2695121, rs56151148, rs2303044 and rs3219281), covering 99.3% of the entire common genetic variability of the *LXRβ *gene were identified and genotyped in the French MONICA adult study (*n *= 2318) and the European adolescent HELENA cross-sectional study (*n *= 1144). *In silico *and *in vitro *functionality studies were performed.

**Results:**

We identified suggestive or significant associations between rs17373080 and the risk of (i) T2DM in HUNT2 (OR = 0.82, *p *= 0.03), (ii) obesity in MONICA (OR = 1.26, *p *= 0.05) and (iii) overweight/obesity in HELENA (OR = 1.59, *p *= 0.002). An intron 4 SNP (rs28514894, a perfect proxy for rs17373080) could potentially create binding sites for hepatic nuclear factor 4 alpha (HNF4α) and nuclear factor 1 (NF1). The *C *allele of rs28514894 was associated with ~1.25-fold higher human *LXRβ *basal promoter activity *in vitro*. However, no differences between alleles in terms of DNA binding and reporter gene transactivation by HNF4α or NF1 were observed.

**Conclusions:**

Our results suggest that rs17373080 in *LXRβ *is associated with T2DM and obesity, maybe *via *altered LXRβ expression.

## Background

Liver X receptors (LXRs) are nuclear receptors involved in control of carbohydrate and lipid homeoastasis as well as inflammation [1-3]. The LXRs consist of two isoforms which share approximately 78% amino acid sequence identity: LXRα, encoded by *NR1H3 *(11p11.2) and LXRβ encoded by *NR1H2 *(19q13.3). High expression of LXRα is restricted to metabolically active tissues, whereas LXRβ is ubiquitously expressed [4]. LXRβ is the only isoform expressed in pancreatic beta cells [5]. LXRs regulate gene expression by binding to specific response elements in the target genes' promoter regions [6-8].

Study of mice lacking one LXR isoform or both has shown important, specific functions of LXRβ in metabolic pathways disturbed in type 2 diabetes mellitus (T2DM) and obesity [9,10]. Firstly, *Lxrβ*^*-/- *^mice exhibit markedly lower glucose tolerance [11] with reduced basal and glucose-stimulated insulin secretion than wild type (wt) mice [5]. The stimulatory effect of LXR agonists on insulin secretion seems to be mediated by regulating both glucose and lipid metabolism in pancreatic beta cells [5,12]. Chronic, increased activation of LXR may contribute to the dysfunction of pancreatic beta cells observed in T2DM [13]. Secondly, *Lxrβ*^*-/- *^mice have less adipose tissue with lower triglyceride (TG) levels but similar insulin sensitivity as wt mice [11,14]. The lack of LXRβ appears to be responsible for the lean phenotype in *Lxrα*^*-/-*^*β*^*-/- *^mice [9].

Therefore, specific LXRβ activation could potentially dissociate the LXR agonists' anti-atherosclerotic and anti-diabetic effects from their hypertriglyceridaemic effects and possibly serve as the basis of a treatment for T2DM [10,15].

We made the hypothesis that the genetic variability of *LXRβ *could be associated with fat mass, glucose metabolism and related phenotypes in humans. In the present study, we identified single nucleotide polymorphisms (SNPs) in the *LXRβ *gene by sequencing DNA from 96 individuals (86 patients with metabolic dysfunctions in glucose and lipid homeostasis and 10 healthy controls). We then performed association studies to determine if the *LXRβ *SNPs were associated with T2DM (in one case-control study), obesity or related phenotypes (in two - adult and adolescent - population based-studies). Finally, the functionality of one SNP was assessed *in silico *and *in vitro*.

## Methods

### The HUNT2 study

The Nord-Trøndelag Health Study 2 (HUNT2 Study) has been described elsewhere http://www.ntnu.edu/hunt[16]. In brief, it was a large (*n *= 65,905), population-based health survey carried out in Norway during 1995-97. Data were obtained from non-fasting blood samples, a clinical examination and questionnaires [17]. From this large sample, 1040 diabetic subjects (942 individuals with self-reported diabetes and 98 individuals with a non-fasting serum glucose level >11.1 mmol/l) were randomly selected excluding type 1 diabetic subjects (*n *= 171) as described [17]. The investigators also selected 2080 age- and gender- matched non-diabetic control subjects with a serum glucose level ≤5.5 mmol/l. DNA samples were available for 2821 subjects (835 T2DM subjects and 1986 controls) [18]. Their characteristics are described in Additional file 1: Table 1. The use of data and DNA materials from these subjects for the present study was approved by the Norwegian Regional Committee for Medical Research Ethics (Southern Region), the Norwegian Social Data Services and the Norwegian Directorate for Health and Social Affairs.

**Table 1:** SNPs detected by sequencing the *LXRβ *gene.

#	rs number	Position on chromosome 19	Position from ATG	Location	Base change	MAF in sequenced individuals (*n *= 96)	MAF in HUNT2 controls (*n *= 1986)	MAF in MONICA (*n *= 2318)	MAF in HELENA-CSS (*n *= 1144)
1	rs79233036^a^	55,569,984	-2692	5' near gene	C>T	0.005 (1 heterozygous subject)	0	-	-
2	rs77094157^a^	55,570,236	-2440	5' near gene	C>T	0.005 (1 heterozygous subject)	NA	-	-
3	rs12972221	55,570,952	-1723	5' near gene	G>T	0.36	NA	-	-
4	**rs17373080**	55,571,336	-1339	5' near gene	C>G	0.37	0.35	0.31	0.32
5	**rs56151148**	55,571,364	-1311	5' near gene	C>T	0.05	0.08	0.09	0.08
6	rs55794952	55,571,487	-1188	5' near gene	G>T	0.005 (1 heterozygous subject)	0.007	-	-
7	**rs2695121**	55,572,553	-122	intron 2	C>T	0.44	0.44	0.43	0.40
8	rs55671147	55,572,866	+192	exon 4	G>A (Glu36Glu)	0.03	NA	-	-
9	rs28514894	55,573,138	+464	intron 4	C>T	0.33	0.35	-	-
10	rs2248949	55,573,981	+1306	intron 6	G>A	0.45	0.44	-	-
11	rs41432149	55,573,984	+1309	intron 6	C>T	0.36	0.35	-	-
12	rs77290536^a^	55,574,080	+1405	exon 7	C>G (Pro252Arg)	0.005 (1 heterozygous subject)	0	-	-
13	rs78105260^a^	55,574,374	+1700	intron 7	A>G	0.005 (1 heterozygous subject)	0.007	-	-
14	rs1405655	55,574,431	+1757	intron 7	C>T	0.36	0.35	-	-
15	rs2303045	55,574,730	+2056	intron 7	C>G	0.35	0.35	-	-
16	rs4802703	55,576,697	+4023	intron 8	A>C	0.40	0.32	-	-
17	**rs2303044**	55,577,013	+4339	intron 8	A>G	0.10	0.07	0.08	0.08
18	rs1052677	55,577,993	+5319	3' UTR	C>G	0.16	0.09	-	-
19	rs75967835^a^	55,578,378	+5704	3' near gene	A>C	0.005 (1 heterozygous subject)	0	-	-
20	**rs3219281**	55,578,899	+6225	3' near gene	C>T	0.15	0.09	0.09	0.10

Positions based on NM_007121 sequence. MAF: minor allele frequency. NA: not available.

The 5 SNPs selected for association studies are indicated in bold.

^a^discovered by gene sequencing in the present study.

### The MONICA study

Participants were recruited as part of the WHO-MONICA population survey conducted from 1995 to 1997 in the Lille Urban Community (Lille, *n *= 1195) and the Haute-Garonne county (Toulouse, *n *= 1182) in France. Individuals aged 35-65 years were randomly selected from electoral rolls (the WHO-MONICA Project protocol) [19]. The study protocol was approved by the appropriate independent ethics committees for each centre. After signing an informed consent form, participants filled out a standard questionnaire. The measurement of anthropometric, clinical and biochemical parameters has been described [20]. Fasting blood samples were drawn into a disodium EDTA tube. DNA samples were available for 2318 individuals. Their characteristics are described in Additional file 1: Table s1.

### The HELENA study

Participants were recruited as part of the HELENA cross-sectional study ("Healthy Lifestyle in Europe by Nutrition in Adolescence-Cross Sectional Study", http://www.helenastudy.com) performed from 2006 to 2007 in 10 centres from 9 European countries as described [21]. The protocol was approved by the appropriate ethics committee in each centre. Written informed consent was obtained from each subject and their legal representatives. The sample included 3865 adolescents (14.8 ± 1.4 y) recruited through their schools which were randomly selected [22]. The measurement of the anthropometric parameters has been described elsewhere [23]. Overweight/obesity was defined as a BMI over the value given by Cole *et al*. [24], corresponding to 25/30 kg/m^2 ^at the age of 18. One third of the classes were randomly selected for fasting blood collection, resulting in a total of 1144 subjects whose characteristics are described in Additional file 1: Table s1. DNA extractions and the measurement of biochemical parameters have also been described [23].

### Gene sequencing

Ten normolipidaemic controls and 86 patients with metabolic syndrome according to the NCEP Expert Panel 2002 definition [25] were recruited at Oslo University Hospital Rikshospitalet and Ulleval, Norway. EDTA blood was collected from the 96 individuals, and genomic DNA was extracted using the MagNaPure LC instrument with DNA isolation kit I (Roche Applied Science, IN, USA). In order to identify as many *LXRβ *variants as possible, the *LXRβ *gene (all exons, exon-intron junctions, 3126 bp 5' of the ATG and 935 bp 3' of the last exon; primer positions in Additional file 1: Table s2) was sequenced in DNA from the 96 individuals, and the SNPs identified were confirmed as described [26] (Table 1).

### Genotyping

In the HUNT2 Study, the SNPs were genotyped on a MASSARRAY system (Sequenom, San Diego, CA, USA) as described elsewhere [26]. The genotyping success rates (GSRs) were over 98% (except for rs77094157, rs12972221 and rs55671147). In the HELENA study, genotyping was performed on an Illumina system using GoldenGate technology. The GSRs were over 99.7%. In the MONICA samples, genotyping was performed using a PCR-RFLP method. Primers and conditions are available on request. The GSRs were over 98%. Two percent of the samples were double genotyped and the concordance rate was 100%. None of the genotype population distributions deviated from the Hardy-Weinberg equilibrium, except for rs2303044 in the MONICA study (*p *= 0.017). However, Hardy-Weinberg equilibrium was respected in the subgroup of normal-weight subjects (*n *= 1076, *p *= 0.14).

### Vectors

The pGL3-*LXRβ *vector was obtained by amplification of the 1922 bp fragment (-201/+1721 bp in reference to the transcription start site) containing part of the promoter through to the end of intron 4 of the *LXRβ *gene in its two allelic forms (*T *and *C*) for rs28514894 (an intermediate step was performed in a TA cloning pCR^®^II vector (Invitrogen)). Primers were 5'-GGCCGCAGGCTCAGAGAAGCG-3' (forward) and 5'-CTGGGGTGGGTAGGTAGAGGC-3' (reverse). Insertion into the pGL3-basic luciferase reporter vector (Promega, Madison, WI) was checked by sequencing. The pCH-NF1A, B, C and X expression vectors were a kind gift from Dr R. Gronostajski (Buffalo, NY, USA). The pCMV6-XL4-HNF4α expression vector was purchased from OriGene Technologies, Inc. (Rockville, MD, USA) (TrueClone SC123863).

### *In silico *analyses

MatInspector version 7.0 [27] (Genomatix Software GmbH, Munich Germany) was used to identify which transcription factor binding sites were affected by the SNPs, using the vertebrate matrix.

### Cell lines and transient transfections

The human JEG3 choriocarcinoma and HepG2 hepatoma cell lines (ATCC) were cultured according to the manufacturer's recommendations. 70% confluent cells in 24-well plates were transfected with 500 ng of pGL3 vector ± 250 ng of HNF4α or NF1 expression vectors with a FuGENE HD (Roche Applied Science):DNA ratio of 5:1 for HepG2 cells and 4:1 for JEG3 cells. Luciferase activities were measured 40 hr after transfection using the Dual Luciferase Assay kit (Promega, Madison, WI). The *firefly *luciferase activity of the pGL3 vectors was normalized against the *renilla *luciferase activity shown by the co-transfected pRL-CMV vector (Promega, Madison, WI, USA).

### Electrophoretic Mobility Shift Assays (EMSAs)

JEG3 cells were transfected in 10 cm dishes with 4 μg NF1 expression vector and 8 μl LipofectAMINE 2000 (Invitrogen). Nuclear extracts were isolated using CelLytic NuCLEAR Extraction Kit (Sigma-Aldrich) with the addition of extra EDTA-free complete proteinase inhibitor cocktail (Roche Applied Science). The oligonucleotides were: rs28514894 T allele 5'-TCCTCTGGCTCTTTGCC**T**GGGGATC-3', rs28514894 C allele 5'-TCCTCTGGCTCTTTGCC**C**GGGGATC-3' (bold letters indicate the SNP), NF1 positive control 5'-GCGGCTCTTGGCCCAAAGCCAGACCT-3' and NF1 negative control 5'-TCCTACTTACACCCTAAGTTTTATC-3' (heavily mutated to hinder NF1 binding). The double-stranded oligonucleotides were radiolabelled and the probes were purified. This was followed by binding reactions and then separation of the protein-DNA complexes from the unbound probes, as described [28]. For supershift assays, 1 μL of anti-haemagglutinin (HA) mouse antibody (Roche Diagnostics GmbH) was pre-incubated with nuclear extracts for 45 minutes at 4°C.

### Statistical analyses

The linkage disequilibrium (LD) between SNPs was evaluated using Haploview [29] and Thesias [30] softwares. All statistical analyses were performed with SAS statistical software (SAS Institute Inc., Cary, NC, USA). Odds ratios were obtained by multivariate logistic regression analyses. To see if the odds ratios were similar between the MONICA studies, the Breslow-Day test of homogeneity of odds ratios was calculated. In order to obtain normal data distributions, log-transformation was used for TG and insulin levels in all samples and for glucose levels in the MONICA sample and an exponential transformation was applied to glucose levels in the HUNT2 study. Inter-group comparisons of quantitative variables were made using a general linear model procedure. Reported p values are nominal and were systematically adjusted for confounding variables. The additive, dominant (and recessive when possible) models were tested but only the best model is actually presented. There was no significant interaction with gender for any of the SNPs in the 3 studies. The threshold for statistical significance was set to *p *≤ 0.01 (0.05/5 tested SNPs).

## Results

### Identification of *LXRβ *SNPs and haplotype blocks

**Figure 1:**
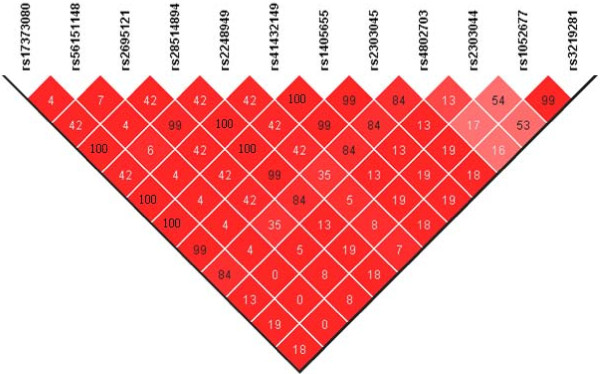
**Linkage disequilibrium map of the 12 LXRβ SNPs (MAF≥0.05) genotyped in the HUNT2 study**. The linkage disequilibrium was analysed using genotypes from control individuals in the HUNT2 study. D' and r^2 ^values were calculated using Haploview software. D' values ranged between 0.88 and 1 and are represented by a red gradation. r^2 ^values (in %) are given within the diamonds.

By sequencing the *LXRβ *gene in 96 individuals 20 SNPs were identified (Table 1). T2DM cases and control subjects from the HUNT2 study were genotyped for these SNPs. Three rare SNPs (rs79233036, rs77290536 and rs75967835) could not be detected (all homozygous subjects). The genotyping of three other SNPs (rs77094157, rs12972221 and rs55671147) suffered from unspecific amplification or low GSR. Rs12972221 was in complete LD with rs17373080 (D' = +1, r^2 ^= 1). Given that the rs77094157 and rs55671147 SNPs had a low minor allele frequency (MAF), they were not further investigated in the present study. Overall, 12 SNPs with a MAF ≥ 0.05 were subsequently analysed. LD between these 12 SNPs was evaluated in control subjects from the HUNT2 study (Figure 1). Three blocks were detected (r^2 ^> 0.80). The first included rs17373080, rs28514894, rs41432149, rs1405655, rs2303045 and rs4802703. Rs17373080 was chosen as a tag-SNP for this block. The second block was composed of rs1052677 and rs3219281 (chosen as a tag-SNP). The third block was composed of rs2248949 and rs2695121 (chosen as a tag-SNP). Thus, the three tag-SNPs (rs17373080, rs3219281 and rs2695121) and the two SNPs (rs56151148 and rs2303044) showing little LD with other SNPs were selected, covering 99.3% of the entire common genetic variability of the *LXRβ *gene.

### Association between the *LXRβ *SNPs and T2DM in HUNT2

We compared the genotype distributions of the 5 SNPs in individuals with T2DM (*n *= 835) and controls (*n *= 1986). No significant distribution differences were found (Table 2). However, when considering a dominant model, there was suggestive evidence for association between rs17373080 and the risk of T2DM. Indeed, subjects carrying the minor G allele had a lower risk of T2DM than CC subjects did (OR [95% CI] = 0.82 [0.69-0.98], *p *= 0.03).

**Table 2:** Genotype distribution for the 5 *LXRβ*; SNPs in individuals with and without T2DM from the HUNT2 study and ORs [95% CI] of T2DM.

	Genotype	Controls (*n *= 1986)		T2DM (*n *= 835)		Crude *p*	Adjusted OR	[95% CI]	Adjusted *p*
						
		*n*	Frequency	*n*	Frequency				
rs17373080	CC	815	0.41	381	0.46				
	CG	934	0.47	367	0.44	0.08	0.82	[0.69-0.98]	**0.03**
	GG	236	0.12	87	0.10				
	Total	1985		835					
									
rs56151148	CC	1655	0.84	684	0.82				
	CT	303	0.15	141	0.17	0.23^a^	1.15	[0.92-1.45]	0.22
	TT	12	0.01	9	0.01				
	Total	1970		834					
									
rs2695121	CC	629	0.32	262	0.31				
	CT	970	0.49	401	0.48	0.79	1.02	[0.85-1.23]	0.80
	TT	382	0.19	170	0.20				
	Total	1981		833					
									
rs2303044	CC	1726	0.87	733	0.88				
	CT	242	0.12	96	0.12	0.43^a^	0.84	[0.65-1.09]	0.20
	TT	12	0.01	2	0.00				
	Total	1980		831					
									
rs3219281	CC	1624	0.83	696	0.84				
	CT	323	0.16	128	0.15	0.27^a^	0.87	[0.69-1.10]	0.25
	TT	21	0.01	4	0.00				
	Total	1968		828					

^a^Fisher exact test.

ORs and *p *values were adjusted for age, gender and BMI.

A dominant model was used to calculate the odds ratios.

Significant *p *values are indicated in bold.

### Association between the *LXRβ *SNPs and obesity in MONICA

Individuals from the MONICA Lille and Toulouse samples (total *n *= 2318) were genotyped for the 5 SNPs. ORs for obesity in minor alleles carriers were homogenous in the 2 samples (Breslow-Day tests). Genotype distributions of the 5 SNPs for obese (BMI≥30 kg/m^2^, *n *= 373) *versus *non-obese (BMI <30 kg/m^2^, *n *= 1945) individuals were compared (Table 3); there were no significant inter-group differences. However, in a dominant model, rs17373080 tended to be associated with the phenotype in question (OR for obesity= 1.26 [1.00-1.59] (*p *= 0.05) for G allele carriers, relative to CC individuals). Haplotype analyses performed with THESIAS did not provide additional information (data not shown).

**Table 3:** Genotype distribution for the 5 *LXRβ*; SNPs in obese and non obese individuals from the MONICA study and ORs [95% CI] of obesity.

	Genotype	Non obese (*n *= 1945)		Obese (*n *= 373)		Crude *p*	Adjusted OR	[95% CI]	Adjusted *p*
						
		*n*	Frequency	*n*	Frequency				
rs17373080	CC	937	0.48	157	0.43				
	CG	804	0.42	166	0.45	0.13	1.26	[1.00-1.59]	**0.05**
	GG	191	0.10	43	0.12				
	Total	1932		366					
									
rs56151148	CC	1595	0.83	314	0.85				
	CT	325	0.17	53	0.14	0.46^a^	0.78	[0.57-1.08]	0.13
	TT	11	0.01	1	0.00				
	Total	1931		368					
									
rs2695121	CC	616	0.32	128	0.35				
	CT	947	0.49	182	0.49	0.30	0.90	[0.71-1.14]	0.39
	TT	370	0.19	59	0.16				
	Total	1933		369					
									
rs2303044	CC	1658	0.85	308	0.83				
	CT	262	0.14	60	0.16	0.41^a^	1.23	[0.91-1.67]	0.18
	TT	20	0.01	3	0.01				
	Total	1940		371					
									
rs3219281	CC	1571	0.83	305	0.82				
	CT	310	0.16	61	0.16	0.77^a^	1.03	[0.76-1.39]	0.85
	TT	19	0.01	5	0.01				
	Total	1900		371					

^a^Fisher exact test. ORs and *p *values were adjusted for age, gender, centre, smoking habit, alcohol consumption and physical activity level. A dominant model was used to calculate the odds ratios. Significant *p *values are indicated in bold.

### Association between the *LXRβ *SNPs and obesity/overweight in HELENA

Adolescents from the HELENA study (*n *= 1144) were genotyped for the 5 SNPs. To gain statistical power, obese (n = 70) and overweight (n = 195) adolescents were pooled. The genotype distributions of the 5 *LXRβ *SNPs between obese/overweight (*n *= 265) and normal-weight (*n *= 879) adolescents were compared (Table 4). As for the MONICA study, the G allele of rs17373080 was associated with a significant higher risk of overweight/obesity (OR = 1.59 [1.19-2.13], *p *= 0.002). Moreover, individuals carrying either the T allele of rs2303044 or the T allele of rs3219281 also had a significant higher risk of obesity (OR = 1.84 [1.27-2.66], *p *= 0.001, and 1.78 [1.25-2.52], *p *= 0.001, respectively), compared with homozygous CC individuals. Haplotype analyses did not provide additional information (data not shown).

**Table 4:** Genotype distribution for the 5 *LXRβ*; SNPs in overweight or obese and normal weight individuals from the HELENA study and ORs [95% CI] of overweight/obesity.

	Genotype	Normal weight (*n *= 879)		Overweight/obese (*n *= 265)		Crude *p*	Adjusted OR	[95% CI]	Adjusted *p*
						
		*n*	Frequency	*n*	Frequency				
rs17373080	CC	427	0.49	102	0.39				
	CG	366	0.42	125	0.48	**0.01**	1.59	[1.19-2.13]	**0.002**
	GG	85	0.10	36	0.14				
	Total	878		263					
									
rs56151148	CC	728	0.83	233	0.88				
	CT	142	0.16	31	0.12	0.13^a^	0.72	[0.47-1.11]	0.14
	TT	9	0.01	1	0.00				
	Total	879		265					
									
rs2695121	CC	301	0.34	106	0.40				
	CT	430	0.49	123	0.47	0.15	0.76	[0.57-1.02]	0.071
	TT	148	0.17	35	0.13				
	Total	879		264					
									
rs2303044	CC	761	0.87	207	0.78				
	CT	111	0.13	53	0.20	**0.003**^a^	1.84	[1.27-2.66]	**0.001**
	TT	5	0.01	4	0.02				
	Total	877		264					
									
rs3219281	CC	732	0.83	200	0.75				
	CT	138	0.16	59	0.22	**0.01**^a^	1.78	[1.25-2.52]	**0.001**
	TT	9	0.01	6	0.02				
	Total	879		265					

^a^Fisher exact test. ORs and *p *values were adjusted for age, gender and centre. A dominant model was used to calculate the odds ratios. Significant *p *values are indicated in bold.

### Association between the *LXRβ *SNPs and obesity- or T2DM-related quantitative phenotypes in HUNT2, MONICA and HELENA

In the three cohorts, we examined associations between the 5 *LXRβ *SNPs and the following obesity-/T2DM-related quantitative phenotypes: BMI, waist circumference, waist-to-hip ratio and plasma glucose and lipid levels.

In control individuals from the HUNT2 study, the G allele of rs17373080 tended to be associated with lower plasma glucose levels (4.96 ± 0.43 in G allele bearers *versus *4.99 ± 0.43 mmol/l in CC subjects, *p *= 0.03). Rs56151148 T allele bearers tended to have lower plasma TG levels (1.62 ± 0.91 *versus *1.69 ± 0.92 mmol/l, *p *= 0.03) compared with CC subjects (Additional file 1: Table s3).

In the MONICA study, there was suggestive evidence for an association between the GG genotype of rs17373080 with a higher waist-to-hip ratio (0.893 ± 0.095 in GG subjects *versus *0.884 ± 0.092 in C allele bearers, *p *= 0.03) (Additional file 1: Table s4). Moreover, rs3219281 T allele carriers had significantly higher plasma TG levels (1.40 ± 1.10 *versus *1.28 ± 0.94 mmol/l, *p *= 0.01) than CC subjects.

In the HELENA study, rs17373080 G allele bearers had higher fasting insulin levels (10.51 ± 6.66 *versus *9.24 ± 4.84 μU/ml, *p *= 0.04) and higher HOMA-IR index (2.36 ± 1.52 *versus *2.07 ± 1.22, *p *= 0.02) than CC subjects (Additional file 1: Table s5). Subjects carrying the rs2695121 T allele had lower fasting insulin levels (9.44 ± 5.12 *versus *10.85 ± 6.98 μU/ml, *p *= 0.008), lower HOMA-IR index (2.10 ± 1.18 *versus *2.45 ± 1.71, *p *= 0.002) and tended to have a lower HOMA-B cell index (136.7 ± 127.3 *versus *153.1 ± 110.9%, *p *= 0.03) compared with CC subjects. The T allele of rs2303044 was associated with a higher BMI (22.1 ± 4.3 in T allele carriers *versus *21.2 ± 3.6 kg/m^2 ^in CC subjects, *p *= 0.006). Lastly, rs3219281 T allele carriers had higher BMI (21.9 ± 4.2 *versus *21.2 ± 3.6 kg/m^2^, *p *= 0.009) and a higher waist circumference (73.4 ± 10.7 *versus *72.0 ± 9.0 cm, *p *= 0.03) than CC subjects.

### Meta-analysis on fat mass indices

We performed a meta-analysis for the 5 SNPs regarding BMI or waist-to-hip ratio. When combining the two adult HUNT2 and MONICA studies (n = 4304), the T allele of rs2303044 was marginally associated with higher waist-to-hip ratio (effect size: +0.0055 ± 0.0028, *p *= 0.05 (heterogeneity *p *= 0.86)). Similar association was detected for the T allele of rs3219281 and waist-to-hip ratio (effect size: +0.0051 ± 0.0024, *p *= 0.03 (heterogeneity *p *= 0.55)). When combining the three HUNT2, MONICA and HELENA studies (n = 5448), the T allele of rs2303044 was significantly associated with higher BMI (effect size: +0.41 ± 0.19 kg/m^2^, *p *= 0.0096 (heterogeneity *p *= 0.39)).

### *In silico *and *in vitro *functional studies of rs28514894

*In silico *analyses with MatInspector software were performed to determine which relevant transcription factor (TF) binding sites were potentially affected by the SNPs. All SNPs in strong LD (r^2 ^= 0.80) with the rs17373080 SNP (i.e. rs28514894, rs41432149, rs1405655, rs2303045 and rs4802703) were tested. The C allele of rs28514894 (located in intron 4 (intron 2 after the translation initiation site)), a perfect proxy for the G allele of 17373080 (r^2 ^= 0.99), could theoretically create a binding site for hepatic nuclear factor 4 alpha (HNF4α) (core similarity = 1.0, matrix similarity = 0.87) or nuclear factor 1 (NF1A, B, C and X) (core similarity = 0.76, matrix similarity = 0.83). Thus, we hypothesized that rs28514894 could modulate the LXRβ transcription level.

**Figure 2:**
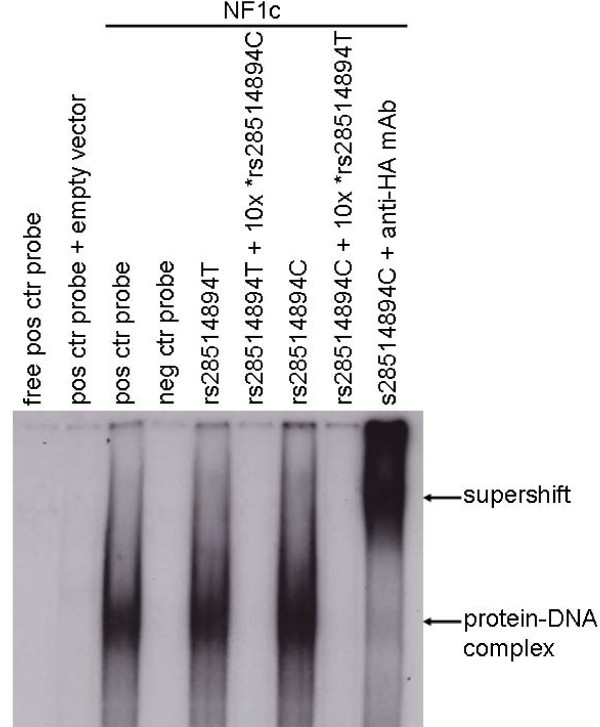
**EMSA experiments showed no difference in TF binding between the rs28514894 SNP alleles, as exemplified by NF1C binding**. EMSA of nuclear extracts (2 μg) isolated from JEG3 cells transfected with the NF1C expression vector revealed a strong specific complex with the LXRβ oligonucleotides encompassing the rs28514894 SNP with both alleles (T or C). The competition experiments were performed using a 10-fold excess of unlabeled oligonucleotides (indicated by *). The upper arrow indicates the supershift with anti-HA-mouse antibody binding to the NF1C protein-oligonucleotide complex and the lower arrow indicates the NF1C protein-oligonucleotide complexes.

To test the functionality of the theoretical NF1 binding sites, we conducted electrophoretic mobility shift assays (EMSAs) with nuclear extracts from JEG3 cells (known not to express NF1) transfected with NF1 expression vectors. Our results showed that the NF1 isoforms could bind similarly to the binding site, regardless of the allele (T or C) (Figure 2). Protein binding to the C allele probe was prevented by adding a 10-fold excess of unlabelled T allele probe and *vice versa*. The specificity of this binding was confirmed, with complete supershifts when adding the anti-HA tag antibody (Figure 2). Nuclear extracts prepared from cells transfected with empty vectors did not bind to any of the probes. Similar experiments were conducted with HNF4α with the same results (data not shown).

**Figure 3:**
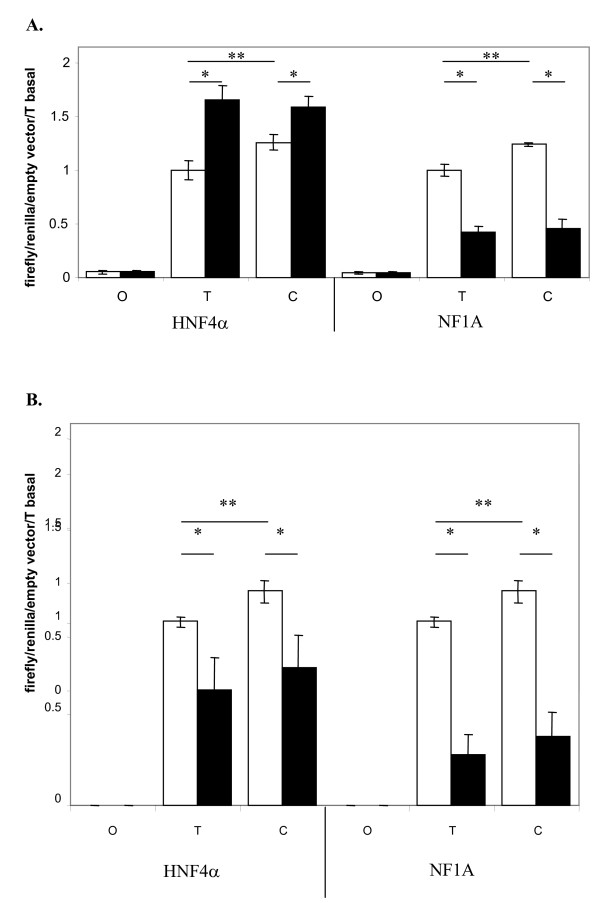
**Functional analyses of the rs28514894 SNP in luciferase reporter gene transfections in HepG2 (A) and JEG3 (B) cells**. HepG2 and JEG3 cells were transiently co-transfected with either the empty basic-pGL3 (O) or the pGL3-LXRβ*T allele or the pGL3-LXRβ*C allele vector with or without an HNF4α or NF1s expression vector. Activity was expressed as fold activity compared with the pGL3-LXRβ*T vector and the empty basic-pGL3 vector. White and black bars represent activities obtained without or with HNF4α or NF1s, respectively. Each bar represents the means ± SEM of at least 3 independent experiments. *Firefly *luciferase activities were normalized against *renilla *luciferase activities. Data were compared using the Kruskal-Wallis non-parametric test. *p < 0.05, ** *p *< 0.01.

The impact of rs28514894 on *LXRβ *promoter activity was also evaluated. We cloned a 1922-bp fragment containing the transcription start site up until the end of intron 4 of the *LXRβ *gene in its two allelic forms (T and C) into the pGL3-basic luciferase reporter vector and thus created the pGL3-*LXRβ *vector. This gene portion was transcriptionally active in both cell lines (~20 fold and ~300 fold compared to the empty pGL3 basic vector in HepG2 and JEG3 cells, respectively) (Figures 3A and 3B). In the basal state, the *LXRβ *fragment displayed ~1.25-fold higher activity (p < 0.05) when carrying the C allele than when carrying the T allele in HepG2 and JEG3 cells. Since rs28514894 is located in a putative binding site for HNF4α and NF1s, the pGL3-*LXRβ *vector was co-transfected with HNF4α or NF1 expression vectors. In HepG2 cells, HNF4α increased *LXRβ*'s activity to a similar extent for the two allelic forms (~1.6 fold for both) (Figure 3A). NF1s repressed *LXRβ *activity by approximately 60% and to a similar extent for the two allelic forms (Figure 3A). Similar results were obtained using the four different NF1s (data not shown) and when using JEG3 cells, except a repressor effect of HNF4α on *LXRβ *in the latter case (Figure 3B).

## Discussion

In the present work, we characterized the impact of the entire common genetic variability of the *LXRβ *gene and searched for associations between 5 tagSNPs and various metabolic phenotypes in 3 independent samples. We report a suggestive association between the rs17373080 minor G allele (representing a 5.7 kbp haplotype block) in *LXRβ *and an approximately 20% lower risk of T2DM in the HUNT2 study. The MONICA study containing only 209 individuals with type 2 diabetes and the HELENA study (adolescents) none, these population samples are not suitable for replication of the association between SNPs and diabetes risk found in HUNT2. However, this result is in line with recent work by Dahlman *et al*., who showed that this allele was associated with a 30% lower risk of T2DM in a study comprising 988 cases and 941 controls [31]. This protective effect can be explained by the lower plasma glucose levels observed in the non-diabetic subjects of the HUNT2 study carrying the G allele. Moreover, we found two *LXRβ *SNPs (rs17373080 and rs2695121) associated with insulin levels and HOMA indexes in adolescents. These associations could reflect LXRβ's role in insulin secretion, since it has been shown that LXRβ activation in pancreatic beta cells increases insulin expression and secretion *via *the SREBP-1 regulated pathway [5,12].

We also showed that the G allele of rs17373080 was associated with higher risk of obesity or overweight in the MONICA and the HELENA studies, respectively. In line with our results, Dahlman *et al*. reported a marginal association (*p *= 0.06) between rs17373080 and the risk of obesity in a study of 559 obese and 438 non-obese individuals [32]. However, they detected no association between rs17373080 and BMI as a continuous trait in 1721 adults [31]. We confirmed this absence of association in a larger sample (n = 5448). Only rs2303044 was significantly associated with BMI when combining the 2 adult and the adolescent studies. The presence of many confounding factors and compensation mechanisms may hide the impact of LXRβ on fat mass.

The opposing effects of the rs17373080 G allele on obesity (deleterious) and T2DM (protective) may appear to be contradictory. However, LXRβ seems to play opposing roles in fat metabolism and glucose homeostasis. *Lxrβ*^*-/- *^mice display lower amounts of adipose tissue on one hand and glucose intolerance (due to impaired glucose-induced insulin secretion) on the other [11]. Furthermore, the effects of the rs17373080 G allele on obesity and T2DM seem to be the opposite of what would be expected from the *Lxrβ*^*-/- *^mouse phenotype [11]. However, this may reflect species differences, as discussed by Dahlman *et al*. [31]. Also, this may reveal differences between a complete gene knock-out and subtle changes like SNPs. The SNPs may induce the recruitment of different co-factors that modify the effect of LXRβ on target genes. For example, if the disease-associated allele creates a binding site for a transactivator, the disease association would be opposite to the effect observed in the *Lxrβ*^*-/- *^mice. Furthermore, epigenetic changes may be involved. Lastly, the fact that LXRβ is expressed in several organs and tissues with different regulatory mechanisms adds to the complexity of the association. Indeed, as shown in our transient transfection experiments, HNF4α activated LXRβ in HepG2 but repressed it in JEG3 cells. The full mechanistic and physiological relevance of the statistical associations found in this and other previous studies should be elucidated in other cell lines expressing the *LXRβ *gene in its various allelic forms.

To the best of our knowledge, no *LXRβ *SNPs have been significantly associated with obesity or T2DM in genome-wide association studies (GWAS). However, due to the very low *p*-value threshold required in GWAS (<10^-8^), nominal associations with *LXRβ *SNPs gene (albeit weak) may have gone unreported. The associations in our present study were probably overestimated, since the number of subjects was lower than in GWAS. Nevertheless, GWAS do not cover the entire genetic variability of each gene. Thus, a candidate gene approach, as in the present study, may help detect associations between SNPs and disorders.

While performing the present study, Dahlman *et al*. [31] found a potential NF1 binding site overlapping rs17373080 using the transcription element search system and showed that NF1 could bind and regulate the expression of the *LXRβ *gene, whatever the allele. In our study, a MatInspector analysis revealed that the minor C allele of rs28514894 (in perfect LD with the minor G allele of rs17373080) could create binding sites for either HNF4α or NF1. We showed that the C allele of rs28514894 was associated with higher *LXRβ *basal promoter activity, which suggested that this allele was associated with higher *LXRβ *mRNA levels. However, we did not observe major difference between the two alleles in terms of DNA binding and transactivation by HNF4α or NF1s. Nevertheless, the inter-allele differences in TF binding and transcriptional activity may be too small to be detected by these methods but can still make a difference in the whole organism over the years - especially if (as would be expected) there are tissue-specific effects.

## Conclusions

In conclusion, our results suggest associations between an *LXRβ *block tagged by rs17373080 and the risks of T2DM and obesity in adults, and the risk of overweight in adolescents, confirming the recent studies by Dahlman *et al*. [31,32]. These findings however must be interpreted with caution and replication in other large population samples and performing meta-analyses are necessary before a link between *LXRβ *gene variability and body weight metabolism can definitely be established. Moreover, understanding the molecular mechanisms behind the *LXRβ *SNP-disorder association will require further experimentation.

## Competing interests

The authors declare that they have no competing interests.

## Authors' contributions

KS participated in study design, coordinated the collaborations, theoretically arranged for gene sequencing and genotyping, prepared samples for gene sequencing, performed the *in silico *and EMSA analysis, wrote and drafted the manuscript. VL carried out the statistical analyses, performed the cloning and the transfection studies and helped to draft the manuscript. KR and ST participated in study design, recruited patients for gene sequencing and drafted the manuscript. PRB arranged gene sequencing and genotyping, prepared the resulting genotyping files and submitted new SNPs to NCBI. KBH, HR, BH, MSN and KIB participated in study design and drafted the manuscript. JF, PA, JR, JV, KW, JRR and JD participated in subject recruitment and helped to the discussion. SL arranged for gene sequencing and genotyping. PMT picked out the T2DM case-control individuals from the HUNT2 study, prepared samples for genotyping, arranged the clinical data file for the HUNT2 individuals and drafted the manuscript. AM participated in the study planning, interpreted the data and participated in writing. HIN initiated the study, participated in study design, arranged the collaborations and drafted the manuscript. All authors read and approved the final manuscript.

## Pre-publication history

The pre-publication history for this paper can be accessed here:

http://www.biomedcentral.com/1471-2350/11/144/prepub

## Supplementary Material

Additional file 1 **Table s1. Clinical characteristics of the study subjects**. This table describes the main clinical characteristics of the 3 studies. Table s2. *LXRβ *gene sequencing primers. This table reports the sequence of the primers used for the sequencing of the *LXRβ *gene. Table s3. Associations for the 5 *LXRβ *SNPs in non-diabetic subjects from HUNT 2. This table reports the associations observed for the 5 SNPs in non-diabetic subjects from HUNT 2. Table s4. Associations for the 5 *LXRβ *SNPs in the MONICA study. This table reports the associations observed for the 5 SNPs in MONICA. Table s5. Associations for the 5 *LXRβ *SNPs in the HELENA study. This table reports the associations observed for the 5 SNPs in HELENA. Click here for file
