# Supplementary material for: Suggestive evidence of associations between liver X receptor β polymorphisms with type 2 diabetes mellitus and obesity in three cohort studies: HUNT2 (Norway), MONICA (France) and HELENA (Europe)
Source: BMC Med Genet. 2010 Oct 12;11:144. doi: 10.1186/1471-2350-11-144 (PMC2958901; doi:10.1186/1471-2350-11-144)
Supplement: Additional file 1 — Table s1. Clinical characteristics of the study subjects. This table describes the main clinical characteristics of the 3 studies. Table s2. LXRβ gene sequencing primers. This table reports the sequence of the primers used for the sequencing of the LXRβ gene. Table s3. Associations for the 5 LXRβ SNPs in non-diabetic subjects from HUNT 2. This table reports the associations observed for the 5 SNPs in non-diabetic subjects from HUNT 2. Table s4. Associations for the 5 LXRβ SNPs in the MONICA study. This table reports the associations observed for the 5 SNPs in MONICA. Table s5. Associations for the 5 LXRβ SNPs in the HELENA study. This table reports the associations observed for the 5 SNPs in HELENA. [file 1471-2350-11-144-S1.PDF]

Additional tables

Additional table 1 Clinical characteristics of the study subjects.

	HUNT2		MONICA	HELENA
	**Controls**	**T2D cases**		
n	1986	835	2318	1144
Men, n (%)	978 (49)	407 (49)	1190 (51)	549 (48)
Age, yrs	65.6 ± 14.3	66.9 ± 13.6	50.7 ± 8.5	14.7 ± 1.4
BMI, kg/m²	26.4 ± 3.9	29.1 ± 5	28.0 ± 4.6	21.3 ± 3.8
Waist circumference, cm	87.9 ± 11.2	96.2 ± 12.5	89.2 ± 13.5	72.4 ± 8.7
Waist/hip ratio	0.86 ± 0.08	0.9 ± 0.08	0.89 ± 0.09	0.79 ± 0.06
Glucose, mmol/L	4.98 ± 0.43	10.2 ± 4.52	5.64 ± 1.41	5.00 ± 0.40
Triglyceride, mmol/L	1.68 ± 0.92	2.5 ± 1.8	1.34 ± 1.51	0.78 ± 0.38
Cholesterol, mmol/L	6.27 ± 1.25	6.1 ± 1.3	5.84 ± 1.05	4.16 ± 0.71
HDL-cholesterol, mmol/I	1.41 ± 0.41	1.2 ± 0.4	1.48 ± 0.46	1.43 ± 0.25

Data are means ± SD or n (%).

Additional table 2 *LXRβ* gene sequencing primers.

Primer pair no:	Forward primer:	Reverse primer:
1	CGATGGGCCTGAGTAGACAT	TGCAAAAATGGTTTCAATAACTTG
2	GGTCCGAGACTGAGCTTTTT	AACCCATTTCCTCGCTTCTT
3	CCAATCACCAAGTTATTGAAACC	GGGAGGAAATGGAAGGAGAG
4	TCCGGTGAGTTTCCAACTTC	TCCAATCCACCTGCTTTTTC
5	GAGGTGGGGTCAGAATGAGA	CAAAGAGCCAGAGGATGGAG
6	GAGGGGTTTAGGAAGGGAGA	GTGGGCAGATGGACCATTA
7	CCCTCCACAACCTTGAGTGT	CTCCAACAGGGATGAGTTGG
8	TGAGCCAAGGAGAGAAAGGA	CTTGGGTGAGACCACGTTTT
9	CACGTGGCAGGTACTCAGTG	CCAGATCTCCGACAGCAGA
10	GTTTCGGGCTGAGGGAGT	ACCCTATGTGCCACCCTTTT
11	GGGCTTCTAGGGACCTCAGT	CCTGGCTTGATCTGTTCTCC

The sequencing was performed between each primer pair.
The primer pair numbers 1-11 are in sequential order within the gene.
The amplicons were overlapping in order to also sequence the DNA covered by the primers.

Additional table 3 Associations for the 5 *LXRβ* SNPs in non-diabetic subjects from HUNT 2.

**rs17373080**<br>(*n*)	CC<br>(807)	CG<br>(915)	GG<br>(231)	Crude *p*	Adjusted *p*<br>G* *vs*. CC
BMI (kg/m²)	26.4 ± 3.9	26.4 ± 4.0	26.1 ± 3.7	0.49	0.55
Waist circumference (cm)	87.6 ± 11.1	88.2 ± 11.2	87.5 ± 10.8	0.43	0.99
Waist/hip ratio	0.854 ± 0.077	0.862 ± 0.081	0.860 ± 0.080	0.12	0.33
Glucose (mmol/l)	4.99 ± 0.43	4.95 ± 0.44	5.00 ± 0.39	0.12	**0.03**
Triglyceride (mmol/l)	1.70 ± 0.92	1.68 ± 0.94	1.66 ± 0.88	0.72	0.28
Cholesterol (mmol/l)	6.27 ± 1.24	6.27 ± 1.25	6.30 ± 1.24	0.96	0.96
HDL-cholesterol (mmol/l)	1.41 ± 0.40	1.42 ± 0.42	1.44 ± 0.41	0.61	0.31
**rs56151148**<br>(*n*)	CC<br>(1628)	CT<br>(299)	TT<br>(12)	Crude *p*	Adjusted *p*<br>T* *vs*. CC
BMI (kg/m²)	26.4 ± 3.8	26.5 ± 4.3	26.0 ± 4.0	0.90	0.67
Waist circumference (cm)	87.9 ± 11.1	88.0 ± 11.5	83.9 ± 8.3	0.46	0.82
Waist/hip ratio	0.858 ± 0.080	0.860 ± 0.079	0.822 ± 0.058	0.29	0.87
Glucose (mmol/l)	4.97 ± 0.42	5.01 ± 0.46	4.98 ± 0.38	0.10	0.07
Triglyceride (mmol/l)	1.69 ± 0.92	1.63 ± 0.92	1.52 ± 0.91	0.24	**0.03**
Cholesterol (mmol/l)	6.28 ± 1.25	6.25 ± 1.21	6.42 ± 1.30	0.84	0.57
HDL-cholesterol (mmol/l)	1.41 ± 0.42	1.44 ± 0.40	1.48 ± 0.32	0.53	0.25
**rs2695121**<br>(*n*)	CC<br>(620)	CT<br>(951)	TT<br>(379)	Crude *p*	Adjusted *p*<br>T* *vs*. CC
BMI (kg/m²)	26.3 ± 4.0	26.4 ± 3.9	26.5 ± 3.7	0.62	0.32
Waist circumference (cm)	88.0 ± 11.1	87.7 ± 11.1	88.1 ± 11.2	0.74	0.51
Waist/hip ratio	0.862 ± 0.082	0.856 ± 0.077	0.859 ± 0.081	0.25	0.75
Glucose (mmol/l)	4.98 ± 0.42	4.97 ± 0.43	4.99 ± 0.43	0.85	0.92
Triglyceride (mmol/l)	1.66 ± 0.94	1.68 ± 0.88	1.73 ± 0.99	0.52	0.19
Cholesterol (mmol/l)	6.32 ± 1.20	6.23 ± 1.27	6.30 ± 1.24	0.36	0.18
HDL-cholesterol (mmol/l)	1.43 ± 0.43	1.42 ± 0.40	1.39 ± 0.42	0.36	0.12
**rs2303044**<br>(*n*)	CC<br>(1702)	CT<br>(235)	TT<br>(12)	Crude *p*	Adjusted *p*<br>T* *vs*. CC
BMI (kg/m²)	26.4 ± 3.9	26.7 ± 4.0	25.9 ± 3.4	0.46	0.52
Waist circumference (cm)	87.8 ± 11.1	88.8 ± 11.4	88.8 ± 6.0	0.42	0.53
Waist/hip ratio	0.858 ± 0.079	0.864 ± 0.084	0.878 ± 0.059	0.40	0.37
Glucose (mmol/l)	4.98 ± 0.44	4.99 ± 0.36	4.88 ± 0.55	0.79	0.73
Triglyceride (mmol/l)	1.68 ± 0.92	1.74 ± 0.98	1.35 ± 0.34	0.49	0.79
Cholesterol (mmol/l)	6.27 ± 1.24	6.34 ± 1.23	5.93 ± 1.25	0.43	0.40
HDL-cholesterol (mmol/l)	1.42 ± 0.42	1.42 ± 0.40	1.33 ± 0.36	0.79	0.64
**rs3219281**<br>(*n*)	CC<br>(1850)	CT<br>(368)	TT<br>(23)	Crude *p*	Adjusted *p*<br>T* *vs*. CC
BMI (kg/m²)	26.4 ± 3.9	26.4 ± 3.9	26.6 ± 4.0	0.97	0.78
Waist circumference (cm)	87.8 ± 11.1	87.9 ± 11.3	91.7 ± 9.3	0.29	0.97
Waist/hip ratio	0.858 ± 0.079	0.860 ± 0.081	0.902 ± 0.072	0.04	0.37
Glucose (mmol/l)	4.98 ± 0.43	4.97 ± 0.41	4.90 ± 0.51	0.57	0.53
Triglyceride (mmol/l)	1.69 ± 0.94	1.70 ± 0.87	1.47 ± 0.49	0.56	0.63
Cholesterol (mmol/l)	6.26 ± 1.24	6.37 ± 1.27	6.18 ± 1.18	0.32	0.07
HDL-cholesterol (mmol/l)	1.41 ± 0.41	1.42 ± 0.44	1.33 ± 0.31	0.59	0.43

Data are means ± SD.
p values were adjusted for age, gender and further adjusted for BMI for biochemical variables.
Significant *p* values are indicated in bold.

Additional table 4 Associations for the 5 *LXRβ* SNPs in the MONICA study.

**rs17373080** (*n*)	CC (1093)	CG (969)	GG (234)	Crude *p*	Adjusted *p* G* *vs.* CC	Adjusted *p* GG *vs.* C*
BMI (kg/m²)	25.9 ± 4.5	26.1 ± 4.8	26.4 ± 4.5	0.42	0.23	0.32
Waist circumference (cm)	89.0 ± 13.4	88.9 ± 13.7	90.2 ± 13.2	0.38	0.40	0.12
Waist/hip ratio	0.885 ± 0.094	0.882 ± 0.090	0.893 ± 0.095	0.43	0.40	**0.03**
Glucose (mmol/l)	5.66 ± 1.51	5.59 ± 1.28	5.64 ± 1.25	0.60	0.35	0.82
Insulin (µU/ml)	11.26 ± 9.04	11.00 ± 6.95	11.39 ± 6.44	0.57	0.46	0.83
HOMA-IR index	2.95 ± 3.13	2.84 ± 2.44	2.96 ± 2.10	0.47	0.28	0.79
HOMA-B cell index (%)	134.0 ± 476.0	119.0 ± 73.8	127.9 ± 118.2	0.64	0.71	0.86
Triglyceride (mmol/l)	1.27 ± 0.90	1.28 ± 0.92	1.43 ± 1.34	0.28	0.40	0.14
HDL-cholesterol (mmol/l)	1.47 ± 0.45	1.50 ± 0.46	1.45 ± 0.44	0.29	0.36	0.26
**rs56151148** (*n*)	CC (1907)	CT (378)	TT (12)	Crude *p*	Adjusted *p* T* *vs.* CC	
BMI (kg/m²)	26.0 ± 4.7	25.9 ± 4.3	26.2 ± 4.8	0.85	0.31	
Waist circumference (cm)	89.1 ± 13.5	88.9 ± 13.4	87.0 ± 11.3	0.82	0.24	
Waist/hip ratio	0.885 ± 0.092	0.883 ± 0.095	0.863 ± 0.102	0.68	0.21	
Glucose (mmol/l)	5.64 ± 1.43	5.59 ± 1.21	5.32 ± 0.78	0.62	0.72	
Insulin (µU/ml)	11.28 ± 8.28	10.53 ± 5.29	11.00 ± 10.15	0.33	0.09	
HOMA-IR index	2.94 ± 2.89	2.69 ± 1.76	2.56 ± 2.45	0.32	0.13	
HOMA-B cell index (%)	129.6 ± 365.8	114.0 ± 61.8	140.3 ± 110.7	0.48	0.15	
Triglyceride (mmol/l)	1.30 ± 0.95	1.26 ± 1.03	1.00 ± 0.41	0.35	0.27	
HDL-cholesterol (mmol/l)	1.48 ± 0.45	1.47 ± 0.47	1.49 ± 0.34	0.85	0.43	
**rs2695121** (*n*)	CC (744)	CT (1128)	TT (428)	Crude *p*	Adjusted *p* T* *vs.* CC	Adjusted *p* TT *vs.* C*
BMI (kg/m²)	26.2 ± 4.7	25.9 ± 4.8	26.0 ± 4.2	0.39	0.25	0.99
Waist circumference (cm)	89.5 ± 13.6	88.6 ± 13.3	88.6 ± 13.6	0.31	0.37	0.23
Waist/hip ratio	0.885 ± 0.092	0.882 ± 0.092	0.889 ± 0.094	0.42	0.58	0.23
Glucose (mmol/l)	5.61 ± 1.29	5.62 ± 1.43	5.69 ± 1.46	0.57	0.51	0.36
Insulin (µU/ml)	11.29 ± 6.55	10.75 ± 6.28	11.37 ± 7.59	0.09	0.30	0.18
HOMA-IR index	2.93 ± 2.48	2.74 ± 1.96	2.98 ± 2.59	0.18	0.56	0.16
HOMA-B cell index (%)	124.6 ± 93.9	114.6 ± 60.1	162.1 ± 757.2	0.09	0.20	0.30
Triglyceride (mmol/l)	1.33 ± 1.11	1.27 ± 0.91	1.28 ± 0.83	0.77	0.51	0.82
HDL-cholesterol (mmol/l)	1.48 ± 0.45	1.48 ± 0.46	1.48 ± 0.46	1.00	0.41	0.95
**rs2303044** (*n*)	CC (1964)	CT (322)	TT (23)	Crude *p*	Adjusted *p* T* *vs.* CC	
BMI (kg/m²)	26.0 ± 4.6	26.2 ± 4.7	25.8 ± 4.8	0.74	0.35	
Waist circumference (cm)	89.1 ± 13.4	89.1 ± 13.7	91.8 ± 16.7	0.63	0.33	
Waist/hip ratio	0.884 ± 0.092	0.884 ± 0.094	0.910 ± 0.096	0.41	0.15	
Glucose (mmol/l)	5.63 ± 1.42	5.59 ± 1.21	5.72 ± 1.16	0.75	0.56	
Insulin (µU/ml)	11.05 ± 7.88	11.69 ± 8.22	13.55 ± 10.88	0.23	0.16	
HOMA-IR index	2.87 ± 2.75	3.03 ± 2.70	3.67 ± 3.42	0.35	0.34	
HOMA-B cell index (%)	126.6 ± 358.3	130.7 ± 121.8	123.2 ± 78.1	0.22	0.84	
Triglyceride (mmol/l)	1.28 ± 0.94	1.31 ± 0.99	1.92 ± 1.88	0.08	0.16	
HDL-cholesterol (mmol/l)	1.48 ± 0.45	1.48 ± 0.47	1.28 ± 0.40	0.11	0.32	
**rs3219281** (*n*)	CC (1875)	CT (371)	TT (24)	Crude *p*	Adjusted *p* T* *vs.* CC	
BMI (kg/m²)	26.0 ± 4.7	26.1 ± 4.5	26.6 ± 4.6	0.78	0.58	
Waist circumference (cm)	89.1 ± 13.6	89.6 ± 13.1	93.4 ± 15.0	0.26	0.26	
Waist/hip ratio	0.884 ± 0.092	0.890 ± 0.093	0.906 ± 0.064	0.26	0.08	
Glucose (mmol/l)	5.63 ± 1.40	5.62 ± 1.26	6.17 ± 1.59	0.07	0.86	
Insulin (µU/ml)	11.05 ± 7.89	11.57 ± 7.95	14.10 ± 8.94	0.07	0.09	
HOMA-IR index	2.87 ± 2.78	3.01 ± 2.60	4.19 ± 3.33	**0.03**	0.13	
HOMA-B cell index (%)	126.6 ± 366.1	127.1 ± 114.9	111.5 ± 62.3	0.50	0.19	
Triglyceride (mmol/l)	1.28 ± 0.94	1.39 ± 1.10	1.52 ± 1.11	**0.03**	**0.01**	
HDL-cholesterol (mmol/l)	1.48 ± 0.46	1.46 ± 0.45	1.22 ± 0.40	**0.02**	0.16	

Data are means ± SD.

p values were adjusted for age, gender, centre, smoking habit, alcohol consumption and physical activity level and further adjusted for BMI for biochemical variables. Significant *p* values are indicated in bold.

Additional table 5 Associations for the 5 *LXRβ* SNPs in the HELENA study.

**rs17373080** (n)	CC (532)	CG (494)	GG (122)	Crude *p*	Adjusted *p* G* *vs*. CC
BMI (kg/m²)	21.2 ± 3.5	21.4 ± 4.0	21.5 ± 3.8	0.45	0.84
Waist circumference (cm)	72.2 ± 9.1	72.4 ± 9.6	72.4 ± 9.3	0.90	0.27
Waist/hip ratio	0.794 ± 0.069	0.796 ± 0.072	0.787 ± 0.060	0.45	0.95
Glucose (mmol/l)	5.01 ± 0.41	5.02 ± 0.39	4.99 ± 0.40	0.58	0.21
Insulin (µU/ml)	9.24 ± 4.84	10.28 ± 6.23	11.39 ± 7.82	**0.03**	**0.04**
HOMA-IR index	2.07 ± 1.22	2.31 ± 1.45	2.55 ± 1.79	**0.01**	**0.02**
HOMA-B cell index (%)	136.2 ± 95.3	147.2 ± 126.6	163.5 ± 129.7	0.06	0.24
Triglyceride (mmol/l)	0.78 ± 0.34	0.79 ± 0.41	0.78 ± 0.37	0.91	0.88
HDL-cholesterol (mmol/l)	1.43 ± 0.26	1.43 ± 0.28	1.45 ± 0.30	0.61	0.28
**rs56151148** (n)	CC (967)	CT (174)	TT (10)	Crude *p*	Adjusted *p* T* vs. CC
BMI (kg/m²)	21.4 ± 3.8	20.9 ± 3.7	20.9 ± 1.9	0.23	0.18
Waist circumference (cm)	72.3 ± 9.5	72.1 ± 8.8	71.8 ± 4.6	0.94	0.68
Waist/hip ratio	0.794 ± 0.073	0.791 ± 0.052	0.789 ± 0.042	0.84	0.73
Glucose (mmol/l)	5.00 ± 0.40	5.01 ± 0.40	4.95 ± 0.37	0.90	0.52
Insulin (µU/ml)	10.33 ± 7.80	10.20 ± 6.88	6.28 ± 1.86	0.07	0.63
HOMA-IR index	2.33 ± 1.94	2.27 ± 1.74	1.34 ± 0.42	0.22	0.56
HOMA-B cell index (%)	144.6 ± 115.2	143.6 ± 106.8	81.5 ± 18.7	0.06	0.97
Triglyceride (mmol/l)	0.78 ± 0.40	0.77 ± 0.34	0.77 ± 0.27	0.94	0.93
HDL-cholesterol (mmol/l)	1.43 ± 0.28	1.42 ± 0.26	1.60 ± 0.39	0.10	0.53
**rs2695121** (n)	CC (411)	CT (555)	TT (184)	Crude *p*	Adjusted *p* T* *vs*. CC
BMI (kg/m²)	21.5 ± 4.0	21.2 ± 3.6	21.1 ± 3.5	0.31	0.13
Waist circumference (cm)	72.8 ± 9.5	71.9 ± 9.4	72.1 ± 8.7	0.39	0.16
Waist/hip ratio	0.795 ± 0.065	0.793 ± 0.076	0.794 ± 0.060	0.93	0.70
Glucose (mmol/l)	5.01 ± 0.42	5.00 ± 0.38	5.00 ± 0.40	0.77	0.27
Insulin (µU/ml)	10.85 ± 6.98	9.48 ± 5.20	9.16 ± 4.68	**0.03**	**0.008**
HOMA-IR index	2.45 ± 1.71	2.11 ± 1.17	2.05 ± 1.12	**0.01**	**0.002**
HOMA-B cell index (%)	153.1 ± 110.9	137.4 ± 113.6	142.9 ± 118.7	**0.03**	**0.03**
Triglyceride (mmol/l)	0.77 ± 0.35	0.79 ± 0.42	0.78 ± 0.36	0.79	0.23
HDL-cholesterol (mmol/l)	1.43 ± 0.28	1.44 ± 0.28	1.41 ± 0.26	0.60	0.66
**rs2303044** (n)	CC (959)	CT (161)	TT (9)	Crude *p*	Adjusted *p* T* *vs*. CC
BMI (kg/m²)	21.2 ± 3.6	22.0 ± 4.4	22.6 ± 3.1	**0.01**	**0.006**
Waist circumference (cm)	72.2 ± 9.2	73.1 ± 10.6	73.4 ± 7.4	0.48	0.18
Waist/hip ratio	0.794 ± 0.065	0.794 ± 0.079	0.757 ± 0.075	0.26	0.96
Glucose (mmol/l)	5.01 ± 0.40	5.02 ± 0.37	5.12 ± 0.45	0.64	0.91
Insulin (µU/ml)	10.19 ± 7.46	10.52 ± 8.98	14.09 ± 5.84	0.06	0.73
HOMA-IR index	2.29 ± 1.86	2.36 ± 2.16	3.36 ± 1.50	**0.04**	0.84
HOMA-B cell index (%)	141.4 ± 105.3	153.9 ± 154.0	191.5 ± 76.2	0.11	0.73
Triglyceride (mmol/l)	0.78 ± 0.39	0.83 ± 0.39	0.86 ± 0.45	0.20	0.29
HDL-cholesterol (mmol/l)	1.43 ± 0.27	1.39 ± 0.28	1.56 ± 0.30	**0.05**	0.53
**rs3219281** (n)	CC (937)	CT (199)	TT (15)	Crude *p*	Adjusted *p* T* *vs*. CC
BMI (kg/m²)	21.2 ± 3.6	21.8 ± 4.3	22.3 ± 3.5	**0.05**	**0.009**
Waist circumference (cm)	72.0 ± 9.0	73.5 ± 11.0	72.2 ± 6.4	0.13	**0.03**
Waist/hip ratio	0.794 ± 0.070	0.794 ± 0.069	0.770 ± 0.048	0.42	0.82
Glucose (mmol/l)	90.85 ± 7.32	91.28 ± 7.13	93.31 ± 5.83	0.32	0.18
Insulin (µU/ml)	10.42 ± 10.51	10.48 ± 8.71	13.60 ± 6.93	**0.05**	0.87
HOMA-IR index	2.28 ± 1.86	2.37 ± 2.11	3.19 ± 1.69	**0.03**	0.74
HOMA-B cell index (%)	141.7 ± 104.7	151.6 ± 149.3	172.5 ± 84.3	0.22	0.61
Triglyceride (mmol/l)	68.66 ± 35.56	69.88 ± 31.95	67.94 ± 26.42	0.62	0.67
HDL-cholesterol (mmol/l)	55.60 ± 10.58	54.02 ± 10.76	57.69 ± 10.96	0.11	0.31

Data are means ± SD.
p values were adjusted for age, gender, centre and further adjusted for BMI for biochemical variables.
Significant *p* values are indicated in bold.
